# Activation and Expansion of Human T-Cells Using Microfluidic Devices

**DOI:** 10.3390/bios15050270

**Published:** 2025-04-25

**Authors:** Ana Belén Peñaherrera-Pazmiño, Gustavo Rosero, Dario Ruarte, Julia Pinter, Karla Vizuete, Maximiliano Perez, Marie Follo, Betiana Lerner, Roland Mertelsmann

**Affiliations:** 1 Centro de Investigación Biomédica (CENBIO), Facultad de Ciencias de la Salud Eugenio Espejo, Universidad UTE, Quito 170527, Ecuador; ana.penaherrera@ute.edu.ec; 2 Ingeniería de Recubrimientos Especiales y Nanoestructuras (IREN) Center, National Technological University, Buenos Aires 1706, Argentina; gustavorosero@gmail.com (G.R.); maxperez@fiu.edu (M.P.); 3 Department of Medicine I, Medical Center—University of Freiburg, Faculty of Medicine, University of Freiburg, 79085 Freiburg im Breisgau, Germany; darioruarte@gmail.com (D.R.); julia_pinter@web.de (J.P.); marie.follo@uniklinik-freiburg.de (M.F.); 4 Centro de Nanociencia y Nanotecnología, Universidad de las Fuerzas Armadas ESPE, Sangolqui 1711031, Ecuador; ksvizuete@espe.edu.ec; 5 Collaborative Research Institute Intelligent Oncology (CRIION), Hermann-Herder-Straße 4, 79104 Freiburg im Breisgau, Germany; 6 Department of Electrical and Computer Engineering, Florida International University, Miami, FL 33174, USA

**Keywords:** microfluidics, suspension cells, T-cell expansion, health

## Abstract

Treatment of cancer patients with autologous T-cells expressing a chimeric antigen receptor (CAR) is one of the most promising therapeutic modalities for hematological malignancy treatment. For this treatment, primary T-cell expansion is needed. Microfluidic technologies can be used to better understand T-cell activation and proliferation. Microfluidics have had a meaningful impact in the way experimental biology and biomedical research are approached in general. Furthermore, microfluidic technology allows the generation of large amounts of data and enables the use of image processing for analysis. However, one of the major technical hurdles involved in growing suspension cells under microfluidic conditions is their immobilization, to avoid washing them out of the microfluidic chip during medium renewal. In this work, we use a multilevel microfluidic chip to successfully capture and immobilize suspension cells. Jurkat cells and T-cells are isolated through traps to microscopically track their development and proliferation after activation over a period of 8 days. The T-cell area of four independent microchannels was compared and there is no statistically significant difference between them (ANOVA *p*-value = 0.976). These multilevel microfluidic chips provide a new method of studying T-cell activation.

## 1. Introduction

Scientists have been working to find methods to isolate and grow different types of cells in order to study how cells behave and develop, and to use this knowledge to create new medical treatments [1]. Different cell types need specific nutrient mixtures in their culture medium to thrive and grow properly in a lab setting [2]. Optimizing the methodology to enable primary T-cell expansion would bring significant advances in individual cell therapy. Cancer patient treatment with autologous T-cells expressing a chimeric antigen receptor (CAR) is one of the most promising therapeutic modalities for hematological malignancy treatment [1] as CARs specifically bind to the antigens expressed on cancer cells. Despite the tremendous success of CAR-T cell therapy against B cell leukemia [3], the CAR-T cell manufacturing process continues to be very complex and expensive due to the variability of the starting material [4]. Therefore, expansion optimization is required to improve the development of CAR-T treatments for solid cancers. In addition, understanding T-cell activation and proliferation contributes to the development of dendritic cell (DC)-based vaccination [5]. Therefore, this research is compatible with sustainable development goal number three.

Further benefits can be derived by miniaturizing these cell cultures, and microfluidics is one way to enable miniaturization. Microfluidics represents the controlled manipulation of fluids inside a channel network [2]. It allows the precise control of the medium that provides nutrients for cells and its composition. Reactant consumption [6] and reaction times [7] can both be reduced. In addition, microfluidic cell culture devices allow lowering the cell population down to a few hundred cells, enabling individual cell [8] or cell clusters to be captured [9,10] and enabling real-time on-chip analysis [11]. However, one of the challenges that microfluidics face is the immobilization of suspension cells such as T-cells during medium renewal [12]. In the literature, Riordon et al. have underlined the extensive potential of combining the use of microfluidics and digital microscopy to acquire image data with automated image analysis for adherent and suspension cells [13]. Indeed, cell retention within grooved microfluidic channels of different widths was explored and computational fluid dynamic modeling was performed to predict shear stresses within the grooves [14]. Furthermore, a microfluidic “Lab-in-a-Trench” (LiaT) platform enabled suspension cells’ capture and retention to analyze glycan density on the surface of individual B lymphocytes [15]. In any case, geometrically aligned sets of triangular pillars have been utilized to isolate circulatory tumor cells (CTCs) based on the strength of cell–cell junctions as clusters that flow at physiological speed [10]. This technology has been denominated the Cluster-Chip and allows isolation of CTCs from unprocessed patient blood samples [10]. However, CTC location among triangular pillars is random and this complicates long-term microscopic monitoring.

In this work, we use a multilevel microfluidic device that captures suspension cells, allowing medium renewal and microscopic examination. Jurkat cells were utilized to establish culture conditions. Once the parameters were set, T-cells were activated and cultured in a multilevel microfluidic device to track their proliferation by image acquisition over a period of 9 days. Image analysis of brightfield microscopy images was performed in order to associate cell size and proliferation, as shown by Renner et al. to be a feasible method to monitor T-cell culture quality [16].

## 2. Materials and Methods

### 2.1. Microfluidic Device Fabrication

**Figure 1:**
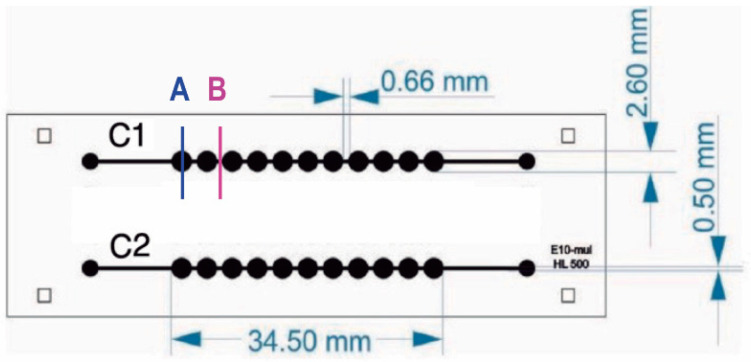
Microfluidic chip design. Microfluidic device has two channels with 11 chambers per channel. The internal volume of each chamber is ~10 uL. Letters A and B indicate well and channel sections, respectively.

A new fabrication methodology developed by the authors enabled the generation of multilevel structures in a practical and one-step method [17,18]. Briefly, microchannel architecture was designed using Layout Editor Software 20241006 (free version Build 6 October 2024). The design of the microfluidic device is depicted in Figure 1. This design was transferred to Photopolymer Flexcel NX Thermal Imaging Layer (TIL) supplied by Eastman Kodak [19]. Sheets of 1270 × 2061 mm^2^ of Flexcel SRH with 1.14 mm thickness [20] were chosen. The organic compounds of the flexographic plate were crosslinked by UVA wavelengths exposure and UVC was applied to stop the reaction. An epoxy resin (Cristal-Tack, Novarchem, Villa Martelli, Argentina) was utilized to make the inverse geometry of the microchannels and form this male mold [21]; the Polydimethylsiloxane (PDMS) replicas with female geometry were obtained. PDMS pre-polymer was mixed with its curing agent SYLGARD 184 (Dow Corning, Midland, MI, USA) at a 10:1 ratio. This degassed PDMS–curing agent mixture was weighted and 3 g were poured on the male mold which was then cured at 40 °C overnight to generate a PDMS replica of 2 mm thickness.

As a result, a 175 ± 0.01 µm depth microchannel was obtained and wells with 468 ± 7.14 µm depth enabled capturing suspension cells.

Then, an additional PDMS slab was made to function as a lid to seal the microchannels from above. An additional 5 g of PDMS–curing agent mixture was poured on a plain epoxy resin mold to obtain a PDMS slab of 5 mm thickness before bonding it to the PDMS replica by exposing both surfaces to oxygen plasma produced by a BD-10A high-frequency generator (Electro-Technic Products, Chicago, IL, USA) to modify the surfaces and form covalent bonds between them. Finally, inlets and outlets were punched into the PDMS slab with a 1 mm biopsy puncher.

**Figure 2:**
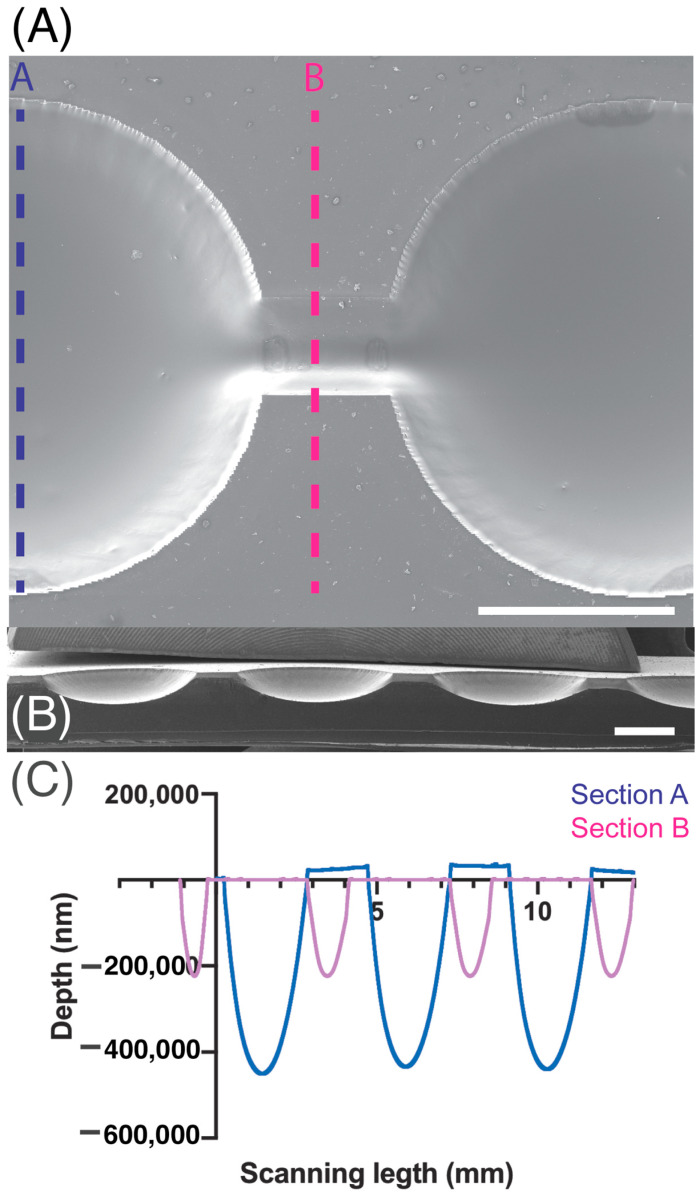
SEM magnification images of microchannel (**A**) top view and (**B**) cross-section. (**C**) Depth measurements acquired by profilometry of section A are indicated in blue and section B measurements are presented in pink. Scale bar: 1 mm.

For characterization, the PDMS replica was imaged through scanning electron microscopy (SEM) and examined with profilometry as presented in Figure 2. SEM images were taken using a 7 kV accelerating voltage with a Scanning electron microscope, SEM Mira 3, Tescan, Brno, Czech Republic.. Previously, the PDMS replica was coated with an approximate 20 nm gold layer with a sputtering evaporator (Quorum Q150R ES, Quorum technologies, Laughton, UK). MIRA TC software version 4.2.24.0 was utilized to perform quantitative measurements, whereas the Dektak XT profilometer from Bruker, Billerica, MA, USA was used to perform profilometry measurements and Vision 64 software was utilized for analysis. Using a tip with a 25 µm radius, linear scans were conducted at a speed of approximately 90 µm·s^−1^ and a sampling rate of 0.01 Hz·mm^−1^. Prior to analysis, the samples were cleaned by first blowing off dust with nitrogen gas, followed by five 10-min ultrasonic cleanings in 70% ethanol. Finally, the samples were dried in a 40 °C oven for 1 h.

### 2.2. Cell Lines and Culture

#### 2.2.1. Jurkat Cell Line

The leukemic cell line Jurkat (ACC-282, Leibniz Institute German Collection of Microorganisms and Cell Cultures, DSMZ) was cultivated using Roswell Park Memorial Institute (RPMI) 1640 medium (Gibco™, Vienna, Austria) supplemented with 10% fetal bovine serum (FBS) and 1% Pen Strep (10,000 Units·mL^−1^ penicillin, 10,000 µg· mL^−1^ streptomycin; Gibco™). Cells were incubated at 37 °C in a humidified atmosphere containing 5% CO_2_ and passaged to a new flask containing fresh medium every two to three days depending on their confluency.

#### 2.2.2. Human T-Cell Isolation and Activation

Blood (20 mL) was extracted from a healthy donor. Since the experiment is an investigation of the blood of a healthy donor volunteer and not a clinical study, there is no Institutional Review Boards Approval required in our institution for this kind of experiment using healthy donors. Peripheral blood mononuclear cells (PBMCs) were separated by centrifugation over a density gradient medium (Anprotec, Bruckberg, Germany). The mononuclear cell layer was collected and washed twice with cold phosphate-buffered saline (PBS). Cells were resuspended in RPMI 1640 medium (Gibco™) containing 10% FBS and 1% Penicillin Streptomycin (Pen Strep) (Life Technologies, Cambridge, UK) and 1 mM EDTA. Easy Sep™ Human T Cell Isolation Kit (STEMCELL technologies, Cologne, Germany) was used following the manufacturer’s instructions. The enriched cell suspension was poured into a new tube. A total of 5 × 10^5^ of the recovered cells were placed in a T-25 culture flask for 48 h, along with 9 mL of 90% RPMI 1640 (1×), Gibco (Life Technologies, Cambridge, UK) supplemented with 10% Fetal Bovine Serum (FBS), 1% Pen Strep (Life Technologies, UK), and 10 ng·mL^−1^ Recombinant Human Interleukin-2 (rh IL-2) (CellGenix, Freiburg, Germany) for stimulation.

#### 2.2.3. Human T-Cell Activation and Proliferation Assay

Both cell lines, Jurkat and the human T lymphocytes, were cultivated in 50 cm^2^ flasks. RPMI 1640 medium (Gibco) supplemented with 10% FBS and 1% Pen Strep (Life Technologies, UK) were utilized as a basic growth medium. Before using human T lymphocytes in the different culture devices, the Jurkat cells were cultured to test seeding and culture procedures within the respective devices. After optimal seeding and culture conditions had been found, human T lymphocytes were then cultured. To determine the concentrations of activation and growth factors for optimal T-cell proliferation, different lectins (phytohemagglutinin (PHA); 1 mg·mL^−1^; Gibco), concanavalin A (Con A; Sigma-Aldrich, St. Louis, MO, USA, C2272-10MG), and the T-cell growth factor interleukin-2 (IL-2; human, 10,000 U (5 µg, 50 mL; Gibco) were tested in different combinations. When the growth medium was renewed, the fresh medium was supplemented with the same growth factor concentrations previously used for the respective assay to maintain the initial growth conditions. However, the Con A effect was not observed. Therefore, to improve T-cell activation, magnetic beads covered with anti-cluster of differentiation 3 (CD3) and anti-CD28 monoclonal antibodies (Dynabeads^®^ Human T-Activator CD3/CD28 for T-Cell Expansion and Activation (11161D); Thermo Fisher Scientific Inc., Karlsruhe, Germany) were used to mimic a more natural physiological environment.

#### 2.2.4. Cell Culture in Microfluidic Devices

**Figure 3:**
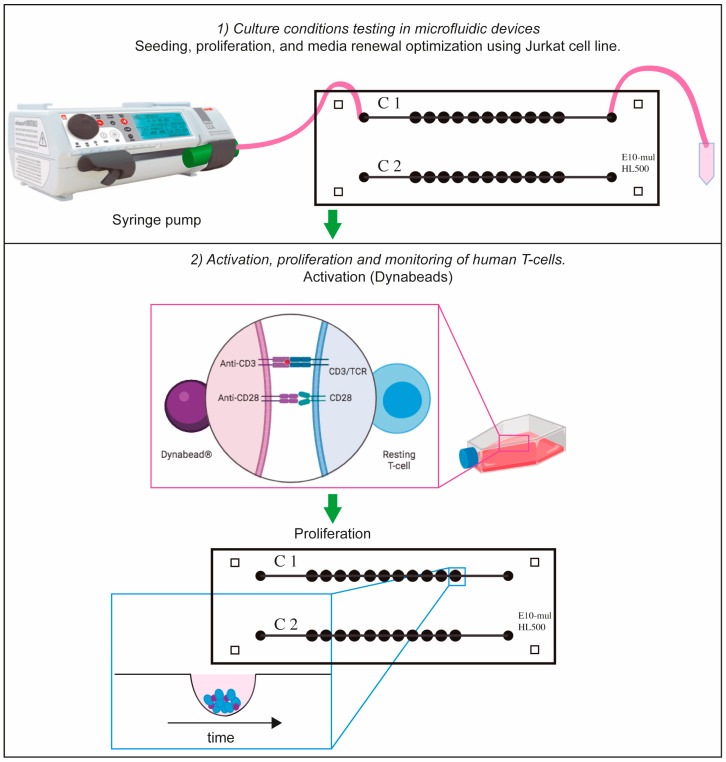
Schematic representation of cell-culturing procedure. Suspension cells (Jurkat or T-cells) were cultured in the microfluidic device by using a syringe pump. In the case of T-cells, they were activated with Dynabeads and they were cultured in T-25 flask for 2 days. After this period of time, they were transferred to the microfluidic device for proliferation tracking. Part of this figure was designed with Biorender.

First, cells were centrifuged at 500 g for five minutes to determine cell concentration. This parameter was measured with an automatic cell counter (TC10 Automated Cell Counter, Bio-Rad Laboratories, Inc., Feldkirchen, Germany) using Trypan Blue dye (0.40%, 1.5 mL; Bio-Rad Laboratories, Inc.). Second, a range of concentrations between 5 × 10^5^ and 1 × 10^6^ cells·mL^−1^ was used to assess the concentration required for optimal growth conditions. After the microfluidic device was sterilized, Jurkat cells were introduced into the microfluidic chip on Day 1 by using a syringe pump (Adox AcTIVA A22 Syringe Infusion Pump; ADOX, Buenos Aires, Argentina) which was placed in an inverted position to prevent cell sedimentation at the syringe walls (Figure 3). Cell morphology was observed for the following eight days (Day 2 to Day 9).

After 2 days, an estimated number of 5 × 10^5^ cells were transferred to the microfluidic device and allowed to settle to the bottom of the microchambers for 24 h. For the Jurkat cell and T-cell experiments, the device was then placed in a Pecon chamber at 37 °C, 5% CO_2_, and monitored from Day 1 to Day 9 in the case of Jurkat cells while T-cells were examined from Day 3 to Day 9 with the Zeiss Axio observer inverted microscope (Carl Zeiss, Oberkochen, Germany) using the EC Plan-Neofluar 2.5×/0.075 and 10×/NA 0.3 objectives (Carl Zeiss, Oberkochen, Germany) to obtain bright-field images.

#### 2.2.5. Cell Viability Assay

A volume of 65 µL of Jurkat cell suspension (3.33 × 10^5^ cells·mL) was administered into each channel of the microfluidic device utilizing a syringe pump (Adox AcTIVA A22 Syringe Infusion Pump; ADOX, Buenos Aires, Argentina) at a flow rate of 26 µL·min over a duration of 3 min. The syringe pump was oriented at a 90-degree angle to mitigate the occurrence of cell sedimentation along the lateral wall of the syringe. The culture medium was replenished on Day 3 at a flow rate of 2 µL·min^−1^ for a period of 35 min for each channel of the microfluidic device. The cell concentration within the culture medium extracted from the outlets was quantified employing the TC10 automatic cell counter, Bio-Rad Laboratories, Inc. Jurkat cells were subjected to microscopic observation, and images were captured from Days 1 to 5 utilizing an inverted Zeiss Axio Observer microscope (Carl Zeiss, Oberkochen, Germany) equipped with the EC Plan-Neofluar 2.5×/0.075 and 10×/0.3 objectives. To avert evaporation, the microfluidic device was situated within a Petri dish that contained water reservoirs. The Petri dish was removed from the incubator to facilitate the transportation of the microfluidic device to the microscope.

The assessment of dead cells was conducted to ascertain the usefulness of the multilevel architectures within the microfluidic device designed for suspension cell cultivation and proliferation. To tackle this concern, the proportion of dead cells was determined by means of image analysis employing propidium iodide (PI) fluorescence, in accordance with the labeling methodology established by Wlodkowic et al. [22] and executed by Zaretsky et al. [23]. Briefly, on Day 5, medium with 0.25 µg·mL^−1^ of PI was flushed into the microchannels to observe the proportion of dead cells. Imaging was conducted utilizing an inverted Zeiss Observer microscope equipped with the appropriate PI filter set. To report the percentage of dead cells, the total cell area for all cells on the bright-field image was considered 100% whereas the area of cells which were labelled with PI was considered as the area of dead cells. The mean area occupied by the Jurkat cells was evaluated from Day 1 to Day 5. The PI positive cells were detected by fluoresce in dark-field images and the percentage of red area was quantified by FIJI-ImageJ.

### 2.3. Image Analysis

Image analysis of the cells cultured in the microfluidic device was performed using FIJI-ImageJ (ImageJ 1.54f) [24]. Cell proliferation was measured by image analysis through a macro developed in FIJI-ImageJ (Supporting Information S1) which determined the area occupied by cells within each image. A total of 350 images of primary T-cells cultured in channels 1 and 2 of two microfluidic devices were acquired during Day 1 to Day 8. Average measurements of 11 wells for each channel are presented, and the standard deviation was calculated according to Equation (1), where x is the sample mean average and n represents the sample size.

(1) $$\sigma =\sqrt{\frac{\sum {\left(x-\bar{x}\right)}^{2}}{\left(n-1\right)}}$$

#### T-Cell Number Estimation

T-cell number was estimated by calculating the volume (V) of the cell cluster which was considered a semi-ellipse and relating it with the T-cell volume at the corresponding day. The volume of the cell cluster was estimated by the following equation:

(2) $$V=\frac{\frac{4}{3}\pi \times a\times b\times c}{2}$$

where V is the volume of the cell cluster and a, b, and c are the lengths of all three semi-axes of the ellipsoid. The lengths of the a and b semi-axes were measured with FIJI-ImageJ while the c semi-axis is the depth of the microchamber.

The number of T-cells (N) was calculated by the following equation.

(3) $$N=\frac{V}{v_{c}}$$

where v_c_ is the volume of a T-cell and V is the volume of the T-cell cluster calculated from Equation (2).

### 2.4. Statistical Analysis

The area occupied by T-cells was measured for each of the 11 wells of each microchannel. A statistically significant difference analysis was performed by using a two-way ANOVA test. All the values are mean ± SD, *p*-value. These values were determined by using GraphPad Prism 10.4.2 (GraphPad Software, Corp.)

## 3. Results

The method used to measure the growth of suspension cells includes three main steps: (1) optimization of cell culture conditions (using Jurkat cells), (2) T-cell activation, and (3) examination of T-cell proliferation. These three results are detailed in the following sections.

### 3.1. Cell Culture Conditions Testing Inside Microfluidic Devices Using Leukemic Jurkat Cell Line

**Figure 4:**
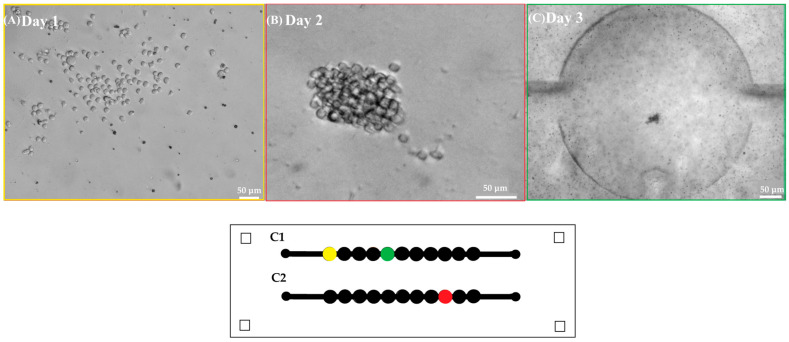
First row shows representative images of (**A**) Jurkat cells in the microfluidic device after seeding (10×) scale bar 50 μm. (**B**) Jurkat cells at the second day of culture (10×) scale bar 50 μm. (**C**) Accumulation of Jurkat cells in the center of a microchamber at the third day of culture (2.5×) scale bar 50 μm. Second row shows a scheme of the microfluidic device where each microchamber location is marked in yellow, red, and green according to the acquired well images of Days 1, 2, and 3.

Approximately, 1.5 × 10^5^ Jurkat cells·mL^−1^ were grown in microfluidic devices with two parallel channels, each containing 11 chambers (Figure 2). To optimize cell seeding and drug treatment procedures, Jurkat cells were seeded in the device using different concentrations of the T-cell-activating lectin concanavalin A (Con A). Cells in the first channel did not receive any stimulating agent while cells in the remaining channel were stimulated with different concentrations of ConA (5 g·mL^−1^, 10 g·mL^−1^ and 15 g·mL^−1^). Rosetta structures were seen in both the control microchannel (without Concanavalin A) and in 5 μg·mL^−1^ Con A microchannels whereas in 10 μg·mL^−1^ and 15 μg·mL^−1^, microchannel rosette structures were not present. Thereon, Con A was not applied. As Jurkat cells produce their growth factor interleukin-2 (IL-2) in an autocrine manner, it was not necessary to add IL-2. To observe cell growth, images of Jurkat cells within the microfluidic device were acquired for 9 days. After seeding, Jurkat cells were dispersed as it can be observed in Figure 4A acquired from microchannel 1, microchamber 1 (yellow). The next day, small accumulations of cells formed in the center of each microchamber as they settled. Figure 4B shows a representative image of cell accumulation located at microchannel 2, microchamber 9 (red). Figure 4C presents a representative image of microchannel 1, microchamber 5 (green) where it can be seen that cells aggregate at the center of the microchamber. This is due to a cell-trapping mechanism generated by the deepest point of each well localized in the center of each microchamber.

**Figure 5:**
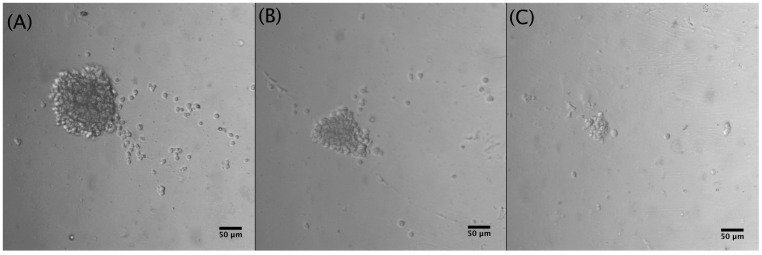
Different Jurkat cell concentrations after 5 days of culture (10×): (**A**) 1 × 10^6^ cells·mL^−1^, cluster diameter: 210 µm; (**B**) 5 × 10^5^ cells·mL^−1^, cluster diameter: 129 µm; (**C**) 2.5 × 10^5^ cells·mL^−1^, cluster diameter: 49 µm.

To identify the best cell-seeding concentration, three different concentrations of Jurkat cells were tested within the microfluidic device: 1 × 10^6^ cells·mL^−1^, 5 × 10^5^ cells·mL^−1^, and 2.5 × 10^5^ cells·mL^−1^. The optimal cell concentration favoring both optical and culture conditions was found to be 5 × 10^5^ cells·mL^−1^ since at this cell concentration. mid-size rosette structures (129 µm) could be detected (Figure 5).

As the cells are not adherent, suspension cells could be washed away if medium renewal is performed at a high flow rate. Therefore, different flow rates were tested and flow rates lower or equal to 2 μL·min^−1^ are suitable for medium renewal.

The viability assay conducted with Jurkat cells demonstrated cell proliferation through a notable augmentation in the mean area occupied by the Jurkat cells from Day 1 to Day 5. Moreover, the region occupied by dead cells was quantified utilizing FIJI-ImageJ, and this measurement was correlated with the area encompassed by the cell cluster at Day 5. (the figure in Supporting Information S3). An average percentage of 0.95% in the wells was calculated, which corresponds to 5.9% of dead cells.

### 3.2. Activation of Human T-Cells

**Figure 6:**
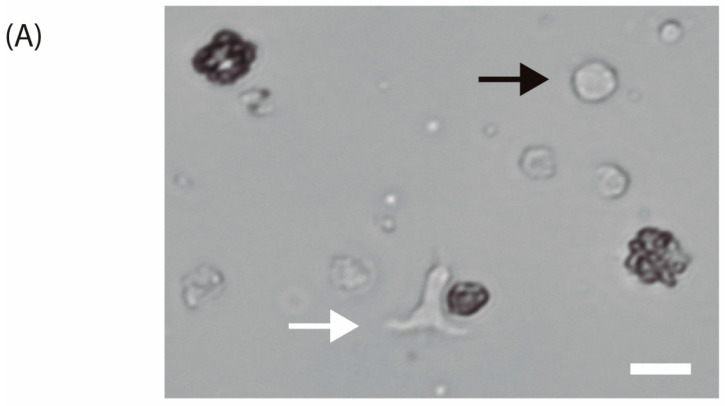

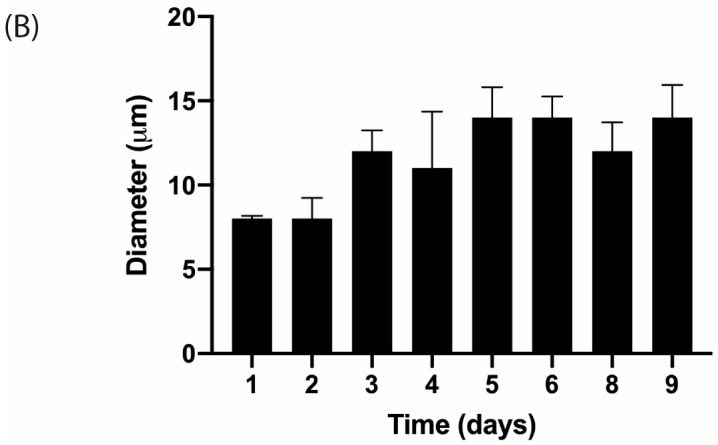
(**A**) Isolated human T-lymphocytes activated with CD3/CD28 beads in vitro conditions after one day of culture (40×). The white arrow indicates an activated T-cell while the black arrow points to a non-activated T-cell. Scale bar: 10 μm. (**B**) T-cell diameter measurements over time with images acquired with 20× objective after activation and 20 ng·mL^−1^ IL-2 stimulation.

T-cell activation is recognized by morphological changes. The change in cellular shape from spherical to elongated can be seen in Figure 6A. Furthermore, the variation of cell diameter over time is depicted in Figure 6B.

### 3.3. Proliferation and Monitoring of Human T-Cells

**Figure 7:**
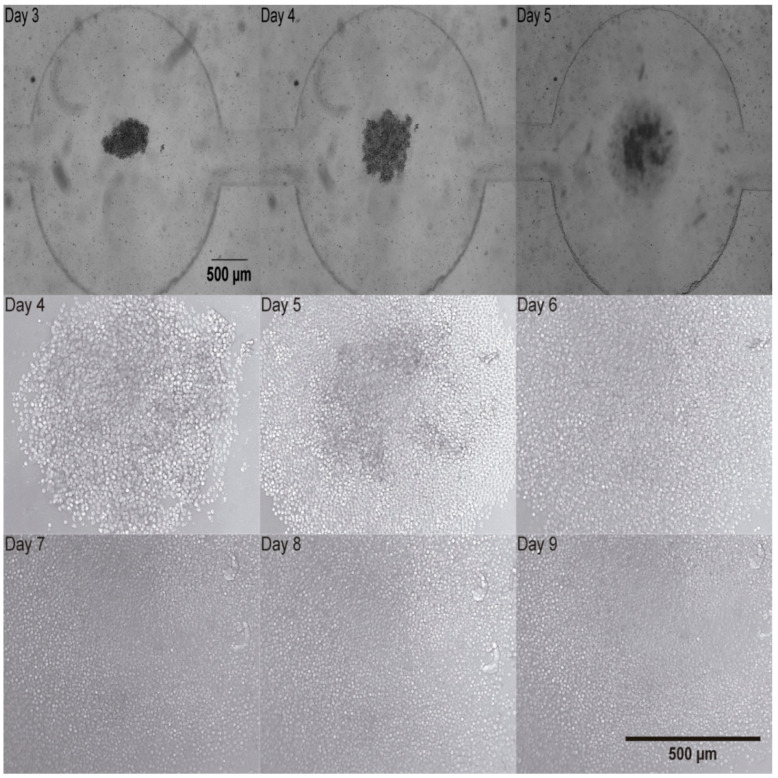
Activated human T-cells trapped at the bottom of the chamber of the microfluidic device from Day 3 to Day 5 (2.5×) upper row. Activated human T-cells development in the microfluidic device from Day 4 to Day 9 (10×). Middle and bottom rows.

To test how healthy human T-cells would react to the miniaturized culture environment found in microfluidic devices, activated human T-cells and magnetic beads were monitored in the microfluidic device for 9 days. After activation with anti CD3/CD28 magnetic beads in T-25 cell culture flasks (Figure 3), activated human T-cells were found to form clusters at the bottom of each microchamber as can be seen in Figure 7. Once cells reach the bottom of the well, they are shielded from shear stress [14]. Medium renewal was performed every 3 days without detrimentally affecting T-cell expansion. Figure 7 shows representative images, depicting cell proliferation.

**Figure 8:**
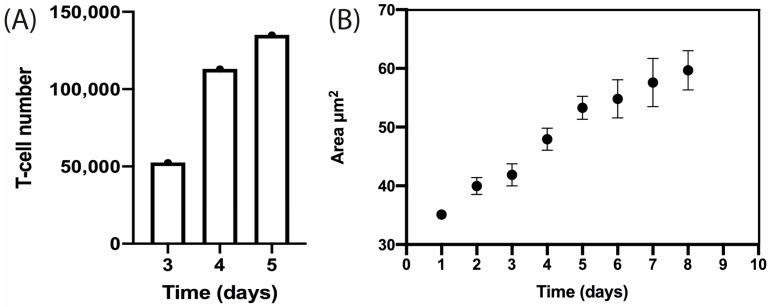
T-cell proliferation within the microfluidic device over time. (**A**) Estimation of T-cell number by semi-ellipse volume calculation from Day 3 to Day 5. (**B**) Estimation of area occupied by activated human T-cells from Day 1 to Day 8. These data correspond to four independent channels. The mean area measurements of 11 wells and standard deviation are shown for each day.

A cluster of T-cells was imaged at 2.5× magnification as depicted in Figure 7. It was trapped in the device and this cluster of cells proliferated as it can be observed in Figure 7 at 10× magnification. T-cell number was estimated by calculating the volume (V) of the cell cluster which was considered a semi-ellipse and relating it with the T-cell volume on the corresponding day (Figure 8A). The area occupied by the T-cells increased from Day 1 to Day 8. The area measurements are described until Day 8 in Figure 8B. These data correspond to four independent channels. The mean area measurements of 11 wells and standard deviation are shown for each day. The ANOVA test showed that there is no statistically significant difference between the four channels, *p*-value = 0.976.

T-cell expansion within the microfluidic device was shown and an image sequence is presented in the Supporting Information (Video S4). T-cell proliferation was measured through image analysis by applying a macro (Supporting Information S1) that performs tasks for cell detection and measurement of cell shape to determine the area occupied by cells and how it increases over time, as described in Figure 8B.

## 4. Discussion

### 4.1. Cell Culture Conditions Testing Inside Microfluidic Devices Using Leukemic Jurkat Cell Line

Before attempting to grow human T-lymphocytes in microfluidic devices, we tested cell concentration, flow rate conditions, and cell viability using the Jurkat cell line, as it shares several physiological aspects with healthy human T-lymphocytes yet tends to be less sensitive to environmental factors and is derived from an immortalized cell line. The selected cell concentration was 5 × 10^5^ cells·mL^−1^ since at this cell concentration mid-size rosette structures could be obtained and longer monitoring could be performed. Higher cell concentrations would produce bigger rosette structures, and the optical field would be full in shorter time.

Flow rates lower or equal to 2 μL·min^−1^ are suitable because higher flow rates could push the rosette out of the well.

Cell viability assay showed that after 5 days of culture, approximately 5.9% of Jurkat cells are dead. This value is in agreement with the values quantified by image analysis under normal culture conditions which correspond to 5.6 ± 2% and 6.7 ± 2% [22]. Therefore, we reasoned that multilevel microstructures enable suspension cell harboring without detrimentally affecting their development.

### 4.2. Activation of Human T-Cells

After we delineated the optimal conditions of seeding concentration and flow rate for medium renewal using the Jurkat cells, we next set our focus on optimizing conditions for activation and expansion of the freshly derived T-cells. There are conventional methods for T-cell activation, (Table 1) such as Stim-R technology (Lyell Immunopharma, USA) [25] that enhances potency, expansion, cytokine production, and higher proliferation potential, T cell TransAct™ [25], human which is used in clinical CAR T-cell manufacturing for in vitro activation and expansion of human T-cells, synthetic antigen-presenting cell scaffolds [26] that physiologically present signals to T-cells and control T-cell expansion. To induce the proliferation of human T-cells, we decided to use magnetic beads which physiologically activate the T-cells. These metallic structures mimic the in vivo interactions which take place between an antigen presenting cell (APC) and a T-cell. After bringing the magnetic beads into contact with the cells at a ratio of 1:2, the human T-cells were seeded in each channel of the microfluidic device. To confirm T-cell activation, we looked for characteristic shape changes, key indicators of this process. When a T-cell interacts with an APC, an immunological synapse (IS) forms between the two cells. This connection is stabilized by adhesion molecules, the T-cell receptor/MHC protein complex, and costimulatory proteins. The formation of the IS triggers the T-cell to transition from round to elongated and/or flattened. This shape change, driven by increased intracellular Ca^2+^ and mitochondrial accumulation at the IS, is linked to the mobilization of F-actin, altering the cell’s cytoskeleton [27]. This elongated shape enables the T-cell to scan for APCs. Upon binding to an APC, the T-cell returns to a round, flattened shape, which implies successful activation [27].

### 4.3. Proliferation and Monitoring of Human T-Cells

T-cell cluster development was microscopically tracked from Day 3 to Day 9 and two parameters were estimated by image analysis. First, T-cell number was calculated by approximating the cluster to a semi-ellipse. Then, it was observed that T-cell number increased from Day 3 to 5 as it is presented in Figure 8A. Second, the area occupied by the cluster was measured and found to increase from Day 4 to Day 7. In the next days, the increase in area could not be measured as the field of view was entirely covered by cells.

This microfluidic approach might impact CAR-T cell therapy manufacturing costs as the volume of reagents is dramatically reduced. In any case, our design can be fabricated in COC for scalability. These advantages could enable manufacturing condition optimization in a microfluidic device before conducting bioreactor experiments. As CAR-T cell therapy cost is exorbitant (over USD 400,000) [30,31], experiments in microfluidic devices which allow for parallelization could definitely decrease costs.

## 5. Conclusions

This study indicates that cell viability was successfully maintained over a period of at least 8 days, as the area occupied by cells could be measured until Day 8, and it increased from Day 1 to Day 8.

The complexities and high expenses involved in trying to rapidly expand human T-cells for their clinical application have made it important to develop a novel, miniaturized method which can be used to study their proliferation ex vivo. In the current study, we have described a novel procedure to successfully culture primary human T-cells in mid/long-term cultures. This study provides a platform that enables microscopically examining the same cluster of T-cells in the time course. Furthermore, manufacturing conditions optimization can be performed in a microfluidic device before conducting bioreactor experiments.

The structure and design of the multilevel microfluidic devices presented here allowed the medium to successfully be renewed, due to a cone-like structure which prevented the accidental washing away of the suspension cells and avoiding detrimental effects. In this way, the microfluidic devices presented here are shown to be suitable candidates for the study of suspension cells. Moreover, our fabrication system is scalable up to 2 m. It enables the creation of a multilevel structure in one step and at a low cost. Although PDMS is suitable for prototyping, once the design passes the proof-of-concept stage, it can be fabricated in cyclic olefin copolymer (COC) which is a stiffer thermoplastic, that can be mass produced, has lower cost, and possesses excellent properties for industrial or clinical applications. Further studies on T-cells such as immunostaining, as well as yeast and bacteria, will be performed in future studies, to continue describing the advantages of these new types of microfluidic devices.

## Figures and Tables

**Table 1:** Comparison of conventional reagents used for T-cell activation.

Reagent	Characteristic	Activation Efficiency	Price (USD/mL)	References
Stim-R technology (Lyell Immunopharma, Seattle, WA, USA)	Synthetic cell biomimetic composed of lipid-coated silica micro-rods that emulate the physiological cell-like presentation of signal molecules to control T-cell properties	Enhanced potency Expansion Cytokine production Persistent cytotoxic function. Improved tumor control in vivo.	Not specified	[25]
T Cell TransAct™, human	Used in clinical CAR-T cell manufacturing. This stimulation reagent is ready-to-use for in vitro activation and expansion of human T-cells. Its polymeric nanomatrix structure ensures gentle and efficient activation of resting T-cells from PBMCs and of enriched T-cell populations, while maintaining high viability.	Provides primary and co-stimulatory signals for optimized and efficient T-cell activation and expansion.	1190	[25,28]
Synthetic antigen-presenting cells (APCs)	Silica microrods loaded with soluble mitogenic cues and coated with liposomes of defined compositions, to form supported lipid bilayers.	Enables several-fold higher polyclonal T-cell expansion and improved antigen-specific enrichment of rare T-cell subpopulations.	Not specified	[26]
Dynabeads^®^ Human T-Activator CD3/CD28 for T Cell Expansion	Beads are 4.5 μm superparamagnetic beads covalently coupled with an optimized combination of anti-human CD3 and anti-human CD28.	Expansion of the T-cells.	477	[29]

## Data Availability

Relevant data were uploaded with the name of the project: Activation and expansion T-cell at the following link: https://figshare.com/account/home#/projects/154484 (accessed on 22 January 2025).

## Supplementary Materials

The following supporting information can be downloaded at https://www.mdpi.com/article/10.3390/bios15050270/s1, Supporting Information S1: Command sequence. Image analysis automation commands performed in FIJI-ImageJ. Supporting Information S2: Correlation between area measurements and cell number. Supporting Information S3: Cell viability assay. Percentage of area occupied by Jurkat cells from Day 1 to Day 5. The last point presents the percentage of area detected for PI positive cells. Video S4: Image sequence of T-cell expansion within the microfluidic device.

## Abbreviations

The following abbreviations are used in this manuscript:

APC	Antigen-presenting cell
CAR	Chimeric Antigen Receptor
CD	Cluster of differentiation
Con A	Concanavalin A
COC	Cyclic olefin copolymer
CTC	Circulatory tumor cells
FBS	Fetal bovine serum
IS	Immunological synapse
PBMC	Peripheral blood mononuclear cells
PBS	Phosphate-buffered saline
PDMS	Polydimethylsiloxane
Pen Strep	Penicillin streptomycin
PI	Propidium iodide
PHA	Phytohemagglutinin
rh IL-2	Recombinant human interleukin-2
RPMI	Roswell Park Memorial Institute Medium
SEM	Scanning electron microscopy
TIL	Thermal imaging layer
