# Supplementary material for: Activation and Expansion of Human T-Cells Using Microfluidic Devices
Source: Biosensors (Basel). 2025 Apr 25;15(5):270. doi: 10.3390/bios15050270 (PMC12110087; doi:10.3390/bios15050270)
Supplement: Supplementary file 1 [file biosensors-15-00270-s001.zip › Supplementary info Biosensors/Supporting information updated.pdf]

Supporting information

S1 Command sequence. Image analysis automation commands performed in FIJI.

Macro for cell area analysis

```
ID = getImageID();
run("Convolve...", "text1=[-1 -1 -1 -1 -1\n-1 -1 -1 -1 -1\n-1 -1 32 -1 -1\n-1 -1 -1 -1 -1\n-1 -1 -1 -1 -1\n] normalize");
run("Enhance Contrast...", "saturated=0.3 equalize");
run("8-bit");
run("Subtract Background...", "rolling=1 sliding disable");
run("Bandpass Filter...", "filter_large=40 filter_small=3 suppress=None tolerance=5 autoscale saturate");
run("Convolve...", "text1=[-1 -1 -1 -1 -1\n-1 -1 -1 -1 -1\n-1 -1 32 -1 -1\n-1 -1 -1 -1 -1\n-1 -1 -1 -1 -1\n] normalize");
run("Threshold...");  // open Threshold tool
  title = "WaitForUserDemo";
  msg = "If necessary, use the \"Threshold\" tool to\nadjust the threshold, then click \"OK\".";
  waitForUser(title, msg);
  selectImage(ID);  //make sure we still have the same image
  getThreshold(lower, upper);
  if (lower==-1)
      exit("Threshold was not set");
      setThreshold(lower, upper);
      run("Convert to Mask");
      run("Close-");
      run("Watershed");
      run("Analyze Particles...", "size=40-Infinity pixel circularity=0.50-1.00 show=Outlines display exclude clear include summarize");
```

S2 Correlation between area measurements and cell number

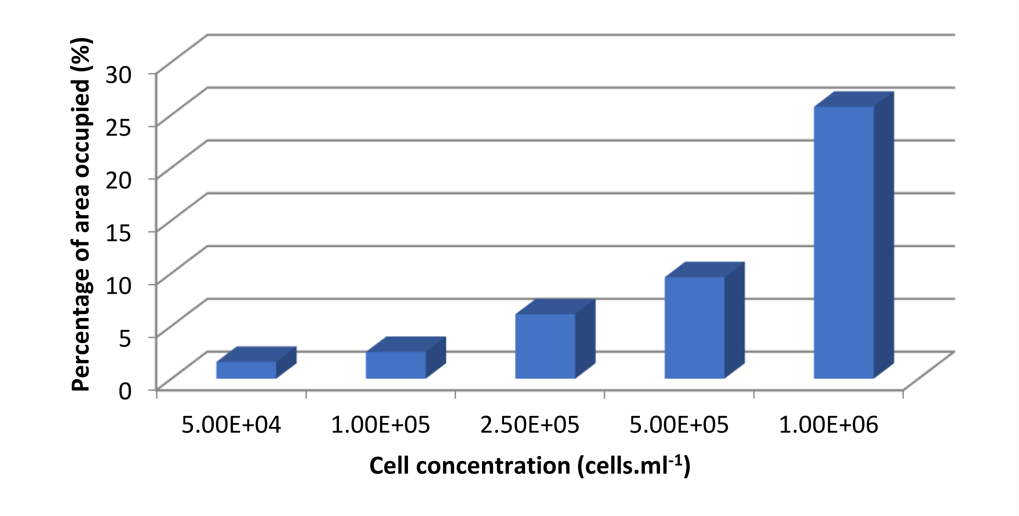

S3 Cell viability assay. Percentage of area occupied by Jurkat cells from day 1 to day 5. The last point presents the percentage of area detected for PI positive cells.

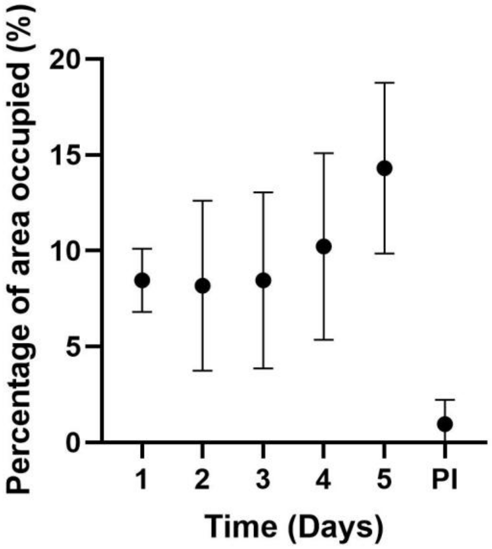

S4 Video. Image sequence of T-cell expansion within the microfluidic device.
